# Genetic and clinical determinants of abdominal aortic diameter: genome-wide association studies, exome array data and Mendelian randomization study

**DOI:** 10.1093/hmg/ddac051

**Published:** 2022-03-02

**Authors:** Eliana Portilla-Fernandez, Derek Klarin, Shih-Jen Hwang, Mary L Biggs, Joshua C Bis, Stefan Weiss, Susanne Rospleszcz, Pradeep Natarajan, Udo Hoffmann, Ian S Rogers, Quynh A Truong, Uwe Völker, Marcus Dörr, Robin Bülow, Michael H Criqui, Matthew Allison, Santhi K Ganesh, Jie Yao, Melanie Waldenberger, Fabian Bamberg, Kenneth M Rice, Jeroen Essers, Daniek M C Kapteijn, Sander W van der Laan, Rob J de Knegt, Mohsen Ghanbari, Janine F Felix, M Arfan Ikram, Maryam Kavousi, Andre G Uitterlinden, Anton J M Roks, A H Jan Danser, Philip S Tsao, Scott M Damrauer, Xiuqing Guo, Jerome I Rotter, Bruce M Psaty, Sekar Kathiresan, Henry Völzke, Annette Peters, Craig Johnson, Konstantin Strauch, Thomas Meitinger, Christopher J O’Donnell, Abbas Dehghan, VA Million Veteran Program

**Affiliations:** Department of Epidemiology, Erasmus University Medical Center, Rotterdam, The Netherlands; Department of Internal Medicine, Division of Vascular Medicine and Pharmacology, Erasmus University Medical Center, Rotterdam, The Netherlands; Center for Genomic Medicine, Massachusetts General Hospital, Harvard Medical School, Boston, MA, USA; Program in Medical and Population Genetics, Broad Institute of MIT and Harvard, Cambridge, MA, USA; Department of Surgery, Massachusetts General Hospital, Boston, MA, USA; Population Sciences Branch, Division of Intramural Research, NHLBI/NIH, Bethesda MD, USA; National Heart Lung and Blood Institute's Intramural Research Program's Framingham Heart Study, Framingham, MA, USA; Cardiovascular Health Research Unit, Department of Medicine, University of Washington, Seattle, WA, USA; Department of Biostatistics, University of Washington, Seattle, WA, USA; Cardiovascular Health Research Unit, Department of Medicine, University of Washington, Seattle, WA, USA; Department of Functional Genomics, Interfaculty Institute for Genetics and Functional Genomics, University Medicine Greifswald, Greifswald, Germany; DZHK (German Centre for Cardiovascular Research), Partner Site Greifswald, Greifswald, Germany; Institute of Epidemiology, Helmholtz Zentrum München – German Research Center for Environmental Health, Neuherberg, Germany; Center for Genomic Medicine, Massachusetts General Hospital, Harvard Medical School, Boston, MA, USA; Program in Medical and Population Genetics, Broad Institute of MIT and Harvard, Cambridge, MA, USA; Cardiovascular Research Center, Massachusetts General Hospital, Boston, MA, USA; Department of Medicine, Brigham and Women's Hospital, Harvard Medical School, Boston, MA, USA; Department of Radiology, Massachusetts General Hospital, Boston, MA, USA; Division of Cardiovascular Medicine, Stanford University, Stanford, CA, USA; Department of Radiology, Weill Cornell Medicine, New York, NY, USA; Department of Functional Genomics, Interfaculty Institute for Genetics and Functional Genomics, University Medicine Greifswald, Greifswald, Germany; DZHK (German Centre for Cardiovascular Research), Partner Site Greifswald, Greifswald, Germany; Department of Internal Medicine, University Medicine Greifswald, Greifswald, Germany; DZHK (German Centre for Cardiovascular Research), Partner Site Greifswald, Greifswald, Germany; Department of Diagnostic Radiology and Neuroradiology, University Medicine Greifswald, Greifswald, Germany; Department of Family Medicine, University of California, San Diego, CA, USA; Department of Family Medicine, University of California, San Diego, CA, USA; Department of Internal Medicine and Human Genetics, University of Michigan, Ann Arbor, MI, USA; The Institute for Translational Genomics and Population Sciences, Department of Pediatrics, The Lundquist Institute for Biomedical Innovation at Harbor-UCLA Medical Center, Torrance, CA, USA; DZHK (German Centre for Cardiovascular Research), Partner Site Greifswald, Greifswald, Germany; Research Unit Molecular Epidemiology, Institute of Epidemiology, Helmholtz Zentrum München – German Research Center for Environmental Health, Neuherberg, Germany; Department of Diagnostic and Interventional Radiology, Faculty of Medicine, University of Freiburg, Freiburg, Germany; Department of Biostatistics, University of Washington, Seattle, WA, USA; Department of Molecular Genetics, Erasmus University Medical Center, Rotterdam, The Netherlands; Department of Radiation Oncology, Erasmus University Medical Center, Rotterdam, The Netherlands; Department of Vascular Surgery, Erasmus University Medical Center, Rotterdam, The Netherlands; Laboratory of Experimental Cardiology, Division Heart and Lungs, University Medical Center Utrecht, Utrecht University, Utrecht, the Netherlands; Laboratory of Clinical Chemistry & Hematology, Division Laboratories, Pharmacy, and Biomedical Genetics, University Medical Center Utrecht, Utrecht University, Utrecht, the Netherlands; Department of Gastroenterology and Hepatology, Erasmus University Medical Center, Rotterdam, the Netherlands; Department of Epidemiology, Erasmus University Medical Center, Rotterdam, The Netherlands; Department of Epidemiology, Erasmus University Medical Center, Rotterdam, The Netherlands; Department of Epidemiology, Erasmus University Medical Center, Rotterdam, The Netherlands; Department of Epidemiology, Erasmus University Medical Center, Rotterdam, The Netherlands; Department of Internal Medicine, Erasmus University Medical Center, Rotterdam, the Netherlands; Department of Internal Medicine, Division of Vascular Medicine and Pharmacology, Erasmus University Medical Center, Rotterdam, The Netherlands; Department of Internal Medicine, Division of Vascular Medicine and Pharmacology, Erasmus University Medical Center, Rotterdam, The Netherlands; Department of Medicine, Division of Cardiovascular Medicine, Stanford University School of Medicine, Stanford, CA, USA; School of Medicine, Stanford University, Stanford, CA, USA; Department of Surgery, Perelman School of Medicine, University of Pennsylvania, Philadelphia, PA, USA; Corporal Michael J. Crescenz VA Medical Center, Philadelphia, PA, USA; The Institute for Translational Genomics and Population Sciences, Department of Pediatrics, The Lundquist Institute for Biomedical Innovation at Harbor-UCLA Medical Center, Torrance, CA, USA; The Institute for Translational Genomics and Population Sciences, Department of Pediatrics, The Lundquist Institute for Biomedical Innovation at Harbor-UCLA Medical Center, Torrance, CA, USA; Cardiovascular Health Research Unit, Department of Medicine, University of Washington, Seattle, WA, USA; Department of Epidemiology, University of Washington, Seattle, WA, USA; Department of Health Services, University of Washington, Seattle, WA, USA; Center for Genomic Medicine, Massachusetts General Hospital, Harvard Medical School, Boston, MA, USA; Program in Medical and Population Genetics, Broad Institute of MIT and Harvard, Cambridge, MA, USA; DZHK (German Centre for Cardiovascular Research), Partner Site Greifswald, Greifswald, Germany; Institute for Community Medicine, University Medicine Greifswald, Greifswald, Germany; Institute of Epidemiology, Helmholtz Zentrum München – German Research Center for Environmental Health, Neuherberg, Germany; Chair of Epidemiology, Institute for Medical Information Processing, Biometry, and Epidemiology, Faculty of Medicine, Ludwig-Maximilians-Universität München, Munich, Germany; German Center for Diabetes Research, Neuherberg, Germany; Collaborative Health Studies Coordinating Center, Department of Biostatistics in the School of Public Health, University of Washington, Seattle, WA, USA; Institute of Genetic Epidemiology, Helmholtz Zentrum München – German Research Center for Environmental Health, Neuherberg, Germany; Chair of Genetic Epidemiology, Institute for Medical Information Processing, Biometry, and Epidemiology, Faculty of Medicine, Ludwig-Maximilians-Universität München, Munich, Germany; Institute of Medical Biostatistics, Epidemiology and Informatics (IMBEI), University Medical Center, Johannes Gutenberg University, Mainz, Germany; DZHK (German Centre for Cardiovascular Research), Partner Site Greifswald, Greifswald, Germany; Institute of Human Genetics, Helmholtz Zentrum München – German Research Center for Environmental Health, Neuherberg, Germany; Institute of Human Genetics, Technische Universität München, München, Germany; Department of Medicine, Brigham and Women's Hospital, Harvard Medical School, Boston, MA, USA; Massachusetts Veterans Epidemiology Research and Information Center (MAVERIC), VA Boston Healthcare System, Boston, MA, USA; Department of Epidemiology and Biostatistics, Imperial College London, London, UK; MRC Centre for Environment and Health, School of Public Health, Imperial College London, London, UK

## Abstract

Progressive dilation of the infrarenal aortic diameter is a consequence of the ageing process and is considered the main determinant of abdominal aortic aneurysm (AAA). We aimed to investigate the genetic and clinical determinants of abdominal aortic diameter (AAD). We conducted a meta-analysis of genome-wide association studies in 10 cohorts (*n* = 13 542) imputed to the 1000 Genome Project reference panel including 12 815 subjects in the discovery phase and 727 subjects [Partners Biobank cohort 1 (PBIO)] as replication. Maximum anterior–posterior diameter of the infrarenal aorta was used as AAD. We also included exome array data (*n* = 14 480) from seven epidemiologic studies. Single-variant and gene-based associations were done using SeqMeta package. A Mendelian randomization analysis was applied to investigate the causal effect of a number of clinical risk factors on AAD. In genome-wide association study (GWAS) on AAD, rs74448815 in the intronic region of LDLRAD4 reached genome-wide significance (beta = −0.02, SE = 0.004, *P*-value = 2.10 × 10^−8^). The association replicated in the PBIO1 cohort (*P*-value = 8.19 × 10^−4^). In exome-array single-variant analysis (*P*-value threshold = 9 × 10^−7^), the lowest *P*-value was found for rs239259 located in SLC22A20 (beta = 0.007, *P*-value = 1.2 × 10^−5^). In the gene-based analysis (*P*-value threshold = 1.85 × 10^−6^), PCSK5 showed an association with AAD (*P*-value = 8.03 × 10^−7^). Furthermore, in Mendelian randomization analyses, we found evidence for genetic association of pulse pressure (beta = −0.003, *P*-value = 0.02), triglycerides (beta = −0.16, *P*-value = 0.008) and height (beta = 0.03, *P*-value < 0.0001), known risk factors for AAA, consistent with a causal association with AAD. Our findings point to new biology as well as highlighting gene regions in mechanisms that have previously been implicated in the genetics of other vascular diseases.

## Introduction

Abdominal aortic diameter (AAD) is an index to measure the widening of abdominal aorta and therefore is a parameter for screening, surveillance and clinical management of abdominal aortic aneurysm (AAA), a focal dilatation of the abdominal aorta (1). An AAD equal or higher than 30 mm is used for diagnosis of AAA, and an AAD ≥ 50–55 mm is an indication for surgical intervention, depending on the patient’s risk factors (2,3). Due to the risk of rupture, AAA is potentially lethal (4) and between 1990 and 2010 the global AAA death rate has increased from 2.49 per 100 000 to 2.78 per 100 000 inhabitants (5). Progressive dilation of the aortic wall is positively associated with ageing, a higher collagen-to-elastin ratio, increased vascular stiffness and high pulse pressure (PP) (6).

Genetic and environmental factors are thought to play an important role on AAD enlargement. Evidence has shown that the variation in the diameter of healthy abdominal aorta is strongly determined by genetic factors with a heritability of 34% (7). Likewise, AAA has been shown to be more than 70% heritable (8) and individuals with a first-degree relative with AAA have a 2-fold higher risk of developing an AAA (9). Initial genetic studies conducted in populations with AAA have identified genes related to abnormal aortic dilatation. These studies have encouraged the implementation of alternative approaches to further deepen investigation of the genetics underlying aortic dilation, including the use of aortic diameter as a continuous trait, leveraging additional power over discrete trait approaches for the limited sample sizes available (10). Here, we aimed to identify genetic factors that affect AAD which was measured in the general population and within a normal range. We applied genome-wide association study (GWAS) to identify (common variants) associated with AAD using data from 10 studies comprising 13 542 participants and exome array analysis to identify (rare variants), using data from seven studies on 14 480 European participants. Further, we implemented Mendelian randomization (MR) analysis to investigate the causal effect of a number of risk factors on AAD.

## Results

### Study sample characteristics

Baseline characteristics for each cohort included in this meta-analysis of genome-wide association studies are summarized in Supplementary Material, Table S1a and b. The discovery panel of genome-wide analysis for common variants included 12 815 participants from nine cohorts. Mean age ranged from 45.1 to 74.9 years and the percentage of males ranged from 27.0 to 57.9. Replication of the GWAS findings was conducted in a sample of 727 individuals from PBIO1 (Partners Biobank cohort 1) cohort. The baseline characteristics of the samples included in the meta-analysis of exome array data are shown in Supplementary Material, Table S2. We analyzed exome array data from 14 480 subjects from seven European cohorts whose mean age ranged from 49.4 to 75.3 years old and the percentage of male population ranged from 42.3 to 58.5.

### Meta-analysis of genome-wide association studies

**Figure 1:**
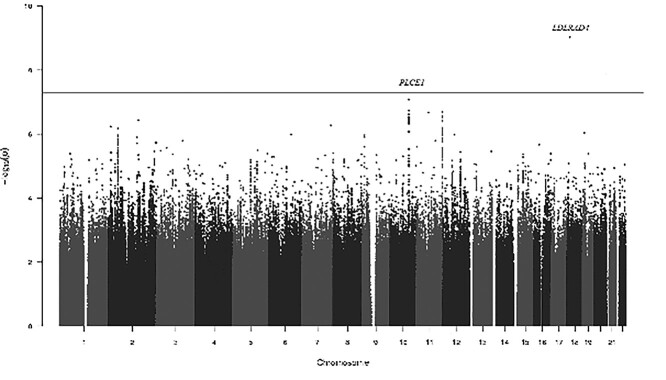
Manhattan plot of meta-analysis of genome-wide association studies on abdominal aortic diameter. Manhattan plot showing the –log-10-transformed *P*-value of SNPs in the GWAS of abdominal aortic diameter. The vertical line indicates the GWAS- significance level (*P*-value < 5 × 10^−8^).

**Figure 2:**
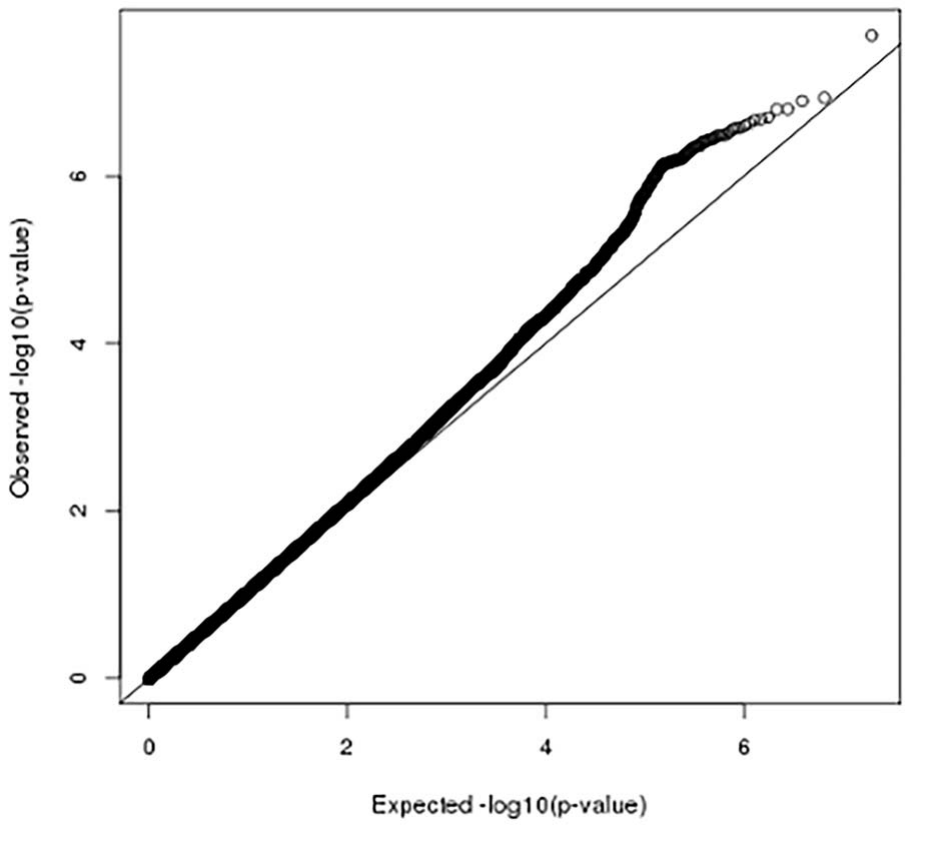
Quantile-quantile plot of genome-wide association studies on abdominal aortic diameter. Vertical and horizontal lines represent expected *P*-values under a null distribution and observed *P*-values, respectively.

Fixed-effect meta-analysis of the summary statistics from each set identified one locus associated with AAD. Figure 1 presents the Manhattan plot of GWAS on AAD. Genome-wide significant and suggestive single nucleotide polymorphisms (SNPs) (5 × 10^−8^ < *P*-value < 5 × 10^−7^) are presented in Table 1. Quantile-quantile plots showed no genomic inflation (α = 1.04) (Fig. 2).

**Table 1:** Associated and suggestive associated loci with abdominal aortic diameter in GWAS

**Locus**	**Chr**	**SNP**	**MAF**	**A1/A2**	**β**	***P*-value combined**	***P*-value in discovery cohort**	***P*-value in replication cohort**
*LDLRAD4*	18	rs74448815	0.04	A/C	-0.02	9.15 × 10^−10^	2.1 × 10^−8^	8.19 × 10^−4^
*PLCE1*	10	rs10882397	0.46	A/C	0.008	8.37 × 10^−8^	2.13 × 10^−7^	0.14
*ADAMTS15 - C11orf44*	11	rs1689231	0.38	C/G	-0.008	3.81 × 10^−7^	2.7 × 10^−7^	0.95

A1: coded allele; A2: non-coded allele; ADAMTS15: ADAM metallopeptidase with thrombospondin type 1 motif 15; Chr: Chromosome; LDLRAD4: low density lipoprotein receptor class A domain containing 4; maf: minor allele frequency, s.e.: standard error.

**Figure 3:**
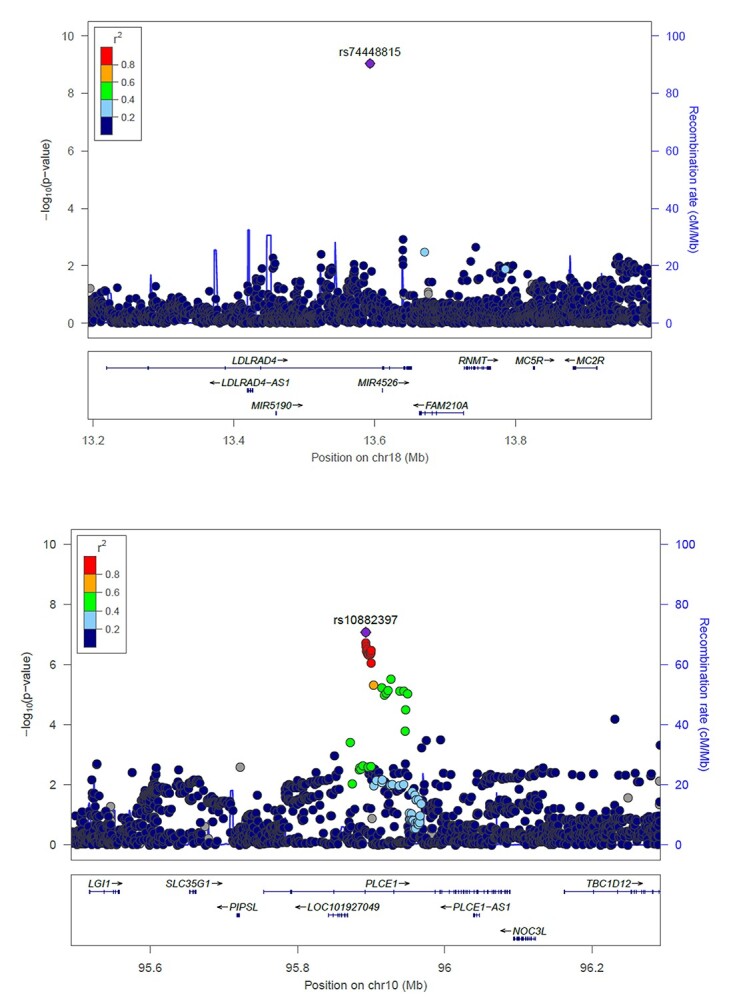
Regional plots showing the association of *LDLRAD4* and *PLCE1* genes with abdominal aortic diameter. *P*-values of genotyped SNPs (circle) and imputed SNPs (square) are plotted (as −log10 *P*-value) against their physical location on chromosome 18p11.21 (**A**) and 10q23.33 (**B**). SNP’s color indicates LD with rs74448815 (A), rs10882397 (B) according to a scale from *r*^2^ = 0 to 1 based on pair-wise *r*^2^ values from 1000 Genomes.

The strongest association was found for rs74448815, located in the intronic region (chr18:13593315) of *LDLRAD4* (Low Density Lipoprotein Receptor Class A Domain Containing 4) (Fig. 3A), where the C allele (MAF [minor allele frequency] = 0.04) was associated with a smaller AAD (beta = −0.026, *P*-value = 2.10 × 10^−8^). This SNP was replicated in PBIO1 cohort (*P*-value = 8.19 × 10^−4^). In addition, the combined meta-analysis showed that the top hit remained genome-wide significant (*P*-value = 9.2 × 10^−10^) (Supplementary Material, Fig. S1) (Table 1).

Furthermore, we found suggestive associations (5 × 10^−8^ < *P*-value < 5 × 10^−7^) for 12 highly correlated SNPs (genetic correlation > 0.80) located in *PLCE1* (Phospholipase C Epsilon 1) (chr10: 95 892 659–95 900 004) (Fig. 3B) gene and six SNPs located in the intergenic region of *ADAMTS15* and *C11orf44* genes (Table 1). In a whole exome sequencing (WES) studies, *ADAMTS15* was found to be linked to intracranial aneurysm in Japanese and German families (11,12). *PLCE1* has been identified as a locus associated with hypertension (13), descending thoracic aneurysm (14) and with stroke in the dbGAP Gene-Trait Associations dataset (http://amp.pharm.mssm.edu/Harmonizome/gene_set/Stroke/dbGAP+Gene-Trait+Associations). Although these variants were not successfully replicated in our analysis, we cannot exclude any indirect effect through risk factors that affect AAD. We further examined the association of AAA-risk loci, previously reported by GWAS and candidate-gene based studies (10,15–21), with AAD (Table 2). *CDKN2BAS1/ANRIL* showed the strongest association with AAD (rs10757274; beta = 0.0058, *P*-value = 1.71 × 10^−4^) (19), and *ERG* (rs2836411; beta = 0.0034, *P*-value = 0.036) (10) and *ADAMTS8* (rs4936098; beta = −0.003, *P*-value = 0.03) (21) were associated with AAD at nominal significance level (Table 2).

**Table 2:** Association of the previously identified risk loci for AAA with abdominal aortic diameter

**SNP**	**Nearest gene**	**A1/A2**	***β*** ^a^	***P*-value** ^b^
***GWAS***				
rs602633	*PSRC1-CELSR2-SORT1*	T/G	0.002	0.32
rs4129267	*IL6R*	T/C	-0.001	0.47
rs10757274	*CDKN2BAS1/ANRIL*	A/G	-0.006	1.71 × 10^−4^
rs10985349	*DAB2IP*	T/C	-0.001	0.58
rs6511720	*LDLR*	T/G	0.001	0.74
rs1795061	*SMYD2*	T/C	0.002	0.35
rs9316871	*LINC00540*	A/G	0.002	0.35
rs3827066	*PCIF1-ZNF335-MMP9*	T/C	0.002	0.44
rs2836411	*ERG*	T/C	0.003	0.036
rs7255	*AC012065.7/LDAH*	C/T	0.003	0.08
rs10023907	*MEPE*	T/C	0.003	0.07
rs3176336	*CDKN1A*	A/T	-0.002	0.21
rs10808546	*RP11-136O12.2/TRIB1*	T/C	-0.001	0.38
rs1412445	*LIPA*	T/C	-0.001	0.46
rs964184	*ZNF259/APOA5*	C/G	0.001	0.52
rs4936098	*ADAMTS8*	A/G	-0.003	0.03
rs35254673	*CRISPLD2*	A/G	0.002	0.29
rs4401144	*CTAGE1*	T/C	0.003	0.07
rs429358	*APOE*	T/C	0.001	0.57
rs11591147	*PCSK9*	T/G	0.004	0.72
rs118039278	*LPA*	A/G	0.001	0.69
rs55958997	*CHRNA3*	A/C	-0.001	0.54
rs73149487	*ABHD16B*	T/G	-0.0008	0.86
***Candidate-gene studies***				
rs599839	*SORT1*	A/G	-0.001	0.46
rs7529229	*IL6R*	T/C	0.001	0.48
rs6743376	*IL1RN*	A/C	0.0001	0.93
rs1542176		T/C	-0.0006	0.68
rs10455872	*LPA*	A/G	-0.002	0.52
rs3798220		T/C	0.007	0.26
rs5186	*AGTR1*	A/C	-0.002	0.17
rs1036095	*TGFBR2*	C/G	0.001	0.53
rs764522		C/G	-0.003	0.08

A1: Coded allele; A2: Non-coded allele; ABHD16B: abhydrolase domain containing 16B; CDKN1A: cyclin dependent kinase inhibitor 1A; CELSR2: cadherin EGF LAG seven-pass G-type receptor 2; CHRNA3: cholinergic receptor nicotinic alpha 3 subunit; Chr: Chromosome; CRISPLD2: cysteine rich secretory protein LCCL domain containing 2; CTAGE1: cutaneous T cell lymphoma-associated antigen 1; DAB2IP: DAB2 interacting protein; ERG: ETS transcription factor ERG; LIPA: lipase A, lysosomal acid type; LPA: lipoprotein(A); MEPE: matrix extracellular phosphoglycoprotein; MMP: matrix metallopeptidase; PCIF1: phosphorylated CTD interacting factor 1; PSRC1: proline and serine rich coiled-coil 1; SMYD2: SET and MYND domain containing 2; SORT1: Sortilin 1; TRIB1: tribbles pseudokinase 1; *P*-value threshold = 0.003.

^a^Beta coefficients from combined AAD meta-analysis.

^b^ *P*-value from combined AAD meta-analysis.

We performed gene-based association analyses using MAGMA to identify tissues and pathways relevant to AAD. Input SNPs were mapped to 18 889 protein-coding genes establishing a genome wide significance *P*-value = 0.05/18884 = 2.6 × 10^−6^. There were no genes associated with AAD at genome-wide significance (Supplementary Material, Table S4). The lowest *P*-value was found for a set of 277 SNPs located in *NUAK1* (NUAK Family Kinase 1) (*P*-value = 4.4 × 10^−6^). Likewise, gene set analysis revealed no significant pathways. Chondrocyte_differentiation, composed of a set of 59 genes, showed the lowest *P*-value = 8.1 × 10^−5^ (Supplementary Material, Table S5).

### SNP heritability and genetic correlation

Our study included data from 13 542 participants from European ancestry. The 1KG intercept was 1.02 (standard error = 0.006). The LD-score calculated SNP Heritability showed an estimate of 0.2 and the mean χ^2^ was 1.07. In addition, a cross-trait LD Score regression method was used to evaluate genome-wide genetic correlation between AAD, AAA, coronary artery disease (CAD) and stroke. We observed a positive significant correlation between AAD and AAA (genetic correlation: 0.45; *P*-value: 0.001), the correlation coefficient between AAD and stroke was 0.1 (*P*-value = 0.3) and between AAD and CAD was 0.06 (*P*-value = 0.4).

### Association of the identified SNPs with gene expression

We found that *LDLRAD4* is highly expressed in several tissues, mainly brain as well as aorta, coronary artery and tibial artery (Supplementary Material, Fig. S2). The isoform ENST00000359446.5, also known as hsa-miR-769-3p, reported the highest expression in arterial tissue [transcripts per million (TPM) = 6.81] in comparison with other isoforms of the gene. Nevertheless, rs74448815 is an intronic SNP without a predicted function and without any proxy SNPs in coding or regulatory regions. Therefore, the effect of this SNP on miRNA regulation is yet to be explored. Likewise, we did not find expression quantitative trait loci (eQTL) associated with this top SNP in both *cis/trans*.

### Meta-analysis of exome array data

The meta-analysis of rare exonic variants associated with AAD was conducted in 14 480 participants from seven European cohorts (Supplementary Material, Table S2). Meta-analysis at the single variant level of exome array variants with MAF > 0.005 (number of variants = 55 461) showed no significant associations with AAD. There was no evidence of genomic inflation (α = 0.57, Supplementary Material, Fig. S3). The lowest *P*-value was observed for rs239259 located in *SLC22A20* (Solute Carrier Family 22 Member 20), chromosomic region 11q13.1, where the T allele has a small effect on aortic dilation (beta = 0.007, *P*-value = 1.20 × 10^−5^). Furthermore, gene-based meta-analysis revealed an association with *PCSK5* (Proprotein Convertase Subtilisin/Kexin Type 5) (*P*-value = 8.03 × 10^−7^), composed by 18 variants. Four variants in the *LDLRAD4* gene were identified for gene-based analysis; however, no association with AAD was observed (*P*-value = 0.21).

### Associations with cardiovascular traits

We further investigated the potential association between one genome-wide significant SNP and five suggestive SNPs found in our meta-analysis, as well as the variant with the lowest *P*-value reported from single variant analysis, with CAD (22) and AAA (21). We found association between our top hit, rs74448815, with a *P*-value = 0.005 reported for CAD (Table 3). In contrast, our top hit was not associated with AAA (*P*-value = 0.95) (Table 3). In addition, two of the suggestive SNPs located in *PLCE1* gene, rs932764 and rs2797983, showed the smallest *P*-value for systolic and diastolic blood pressure (DBP) (23) and AAA, respectively (Table 3).

**Table 3:** Association of the identified AAD-SNPs with vascular traits

**SNP**	**Nearest gene**	***P*-value**	**Trait**
rs74448815	*LDLRAD4*	0.95	AAA
		0.005	CAD
rs932764	*PLCE1*	6.88 × 10^−17^	SBP
		6.28 × 10^−10^	DBP
rs2797983		1.34 × 10^−4^	AAA

AAA: abdominal aortic aneurysm; CAD: coronary artery disease; DBP: diastolic blood pressure; LDLRAD4: low-density lipoprotein receptor class A domain containing 4; SBP: systolic blood pressure.

### Genetic risk score and Mendelian randomization analysis

We tested the association between the polygenic risk score, constructed from AAA-related SNPs, and AAD in Rotterdam Study (RS). We found no association between the genetic score and AAD (beta = 0.0004, *P*-value = 0.09).

We further examined the potential causal association between AAA risk factors and AAD, including systolic blood pressure (SBP), DBP, PP, smoking, lipid traits [low-density lipoprotein (LDL), high-density lipoprotein (HDL) and triglycerides (TG)], height and body mass index (BMI). We examined 104 SNPs reported for SBP (Supplementary Material, Table S6), 139 SNPs associated with DBP (Supplementary Material, Table S7), 109 SNPs reported for PP (Supplementary Material, Table S8), 124 variants associated with smoking (Supplementary Material, Table S9), 66 SNPs for LDL (Supplementary Material, Table S10), 39 SNPs for HDL (Supplementary Material, Table S11), 35 SNPs for TG (Supplementary Material, Table S12), 134 SNPs associated with height (Supplementary Material, Table S13) and 73 SNPs found for BMI (Supplementary Material, Table S14).

Results from the conventional and sensitivity MR analyses are shown in Table 4. Penalized inverse-variance weighting (IVW) estimates suggested that a 1-SD genetically elevated height is associated with AAD (IVW estimate = 0.03, *P*-value < 0.0001) (Supplementary Material, Fig. S3). Furthermore, we found evidence to support that 1-SD genetically increased PP demonstrated a significant association with infrarenal aortic diameter (IVW estimate = −0.003, *P*-value = 0.02) (Supplementary Material, Fig. S4). For lipid traits, we conducted a conventional 2-sample MR analysis to assess for evidence of a causal role of LDL-cholesterol on AAD. IVW estimates were not significant for any of the each lipid fractions [LDL: IVW = −0.005, *P*-value = 0.2 (Supplementary Material, Fig. S5); HDL: IVW = −0.002, *P*-value = 0.9 (Supplementary Material, Fig. S6); TG: IVW = −0.02, *P*-value = 0.2]. Nevertheless, MR-Egger estimate showed that a 1-SD genetically increase in TG was significantly associated with a smaller AAD (beta = −0.16, *P*-value = 0.008, Supplementary Material, Fig. S7). We did not find evidence for causal association of systolic blood pressure (IVW estimate = 0.0001, *P*-value for IVW = 0.8, Supplementary Material, Fig. S8), DBP (IVW estimate = 0.001, *P*-value for IVW = 0.5, Supplementary Material, Fig. S9), smoking (IVW estimate = 0.02, *P*-value for IVW = 0.3, Supplementary Material, Fig. S10) and BMI (IVW estimate = −0.005, *P*-value for IVW = 0.1, Supplementary Material, Fig. S11) with AAD (Table 4).

## Discussion

The present study is the largest genome-wide study including both common and rare variants with AAD to date, utilizing GWAS data of 13 542 European ancestry participants from 10 studies and exome array data from seven studies with 14 480 subjects. We identified two novel loci at *LDLRAD4* and *PCSK5* associated with AAD. At least one out of the 18 risk loci that were previously reported for AAA was also nominally associated with AAD. Furthermore, we showed that height and PP, known clinical risk factors for AAA, may be causally associated with AAD.

**Table 4:** Mendelian randomization results for each risk factor

**Systolic blood pressure**			
**Method**	**Estimate**	***P*-value**	***P*-value heterogeneity (IVW)**
IVW	−0.001	0.2	0.04
MR-Egger	0.001	0.8	-
MR-Egger (intercept)	0	0.6	-
Weighted median	0.0001	0.9	-
**Diastolic blood pressure**			
IVW	−0.001	0.6	0.2
MR-Egger	−0.002	0.5	-
MR-Egger (intercept)	0	0.6	-
Weighted median	0.001	0.8	-
**Pulse pressure**			
IVW	−0.003	0.02	0.02
MR-Egger	−0.009	0.04	-
MR-Egger (intercept)	0	0.1	-
Weighted median	−0.003	0.06	-
**Smoking**			
IVW	0.02	0.3	0.03
MR-Egger	0.01	0.9	-
MR-Egger (intercept)	0	0.9	-
Weighted median	−0.006	0.8	-
**LDL**			
IVW	−0.005	0.2	0.04
MR-Egger	−0.004	0.5	-
MR-Egger (intercept)	0.0001	0.8	-
Weighted median	−0.006	0.3	-
**HDL**			
IVW	−0.002	0.9	0.002
MR-Egger	−0.02	08	-
MR-Egger (intercept)	0	0.8	-
Weighted median	0.009	0.7	-
**Triglycerides**			
IVW	−0.02	0.2	0.2
MR-Egger	−0.16	0.008	-
MR-Egger (intercept)	0.003	0.01	-
Weighted median	−0.03	0.1	-
**Height**			
IVW	0.03	0.0001	0.05
MR-Egger	0.07	0.0001	-
MR-Egger (intercept)	−0.001	0.008	-
Weighted median	0.03	0.0001	-
**BMI**			
IVW	0.01	0.0001	0.06
MR-Egger	0.004	0.3	-
MR-Egger (intercept)	0.001	0.001	-
Weighted median	0.004	0.26	-

BMI: body mass index; HDL: high-density lipoprotein; IVW: inverse-variance weighting; LDL: low-density lipoprotein; MR: Mendelian randomization.

Our GWAS identified *LDLRAD4* to be associated with AAD. Prior studies have demonstrated an association of genetic variants in *LDLRAD4* with schizophrenia bipolar disorder (24,25), and a low-frequency variant (rs8096897) in this gene showed evidence of association with systolic blood pressure (26). This gene has also been described as a prognostic indicator in primary gastrointestinal stromal tumors (27). *LDLRAD4*, also known as *C18orf1*, is involved in the downregulation of transforming growth factor-β (TGF-β) by interacting with downstream effectors SMAD2 (SMAD Family Member) and SMAD3 via its SIM (SIM BHLH Transcription Factor) domain (28). In the canonical TGF-β pathway, ZFYVE9/SMAD Anchor for Receptor Activation (SARA) (Zinc Finger FYVE-Type Containing 9) recruits the intracellular signal transducer and transcriptional modulators SMAD2 and SMAD3 to the TGF-β receptor. After phosphorylation by the receptor, SMAD2 and SMAD3 then form a heteromeric complex with SMAD4 that translocates to the nucleus to regulate transcription. Through interaction with SMAD2 and SMAD3, LDLRAD4 may compete with ZFYVE9 and SMAD4 and prevent propagation of the intracellular signal.

TGF-β plays a crucial role during embryogenesis, and its downregulation in adult life contributes to the development of vascular disorders, including AAA (29). Heterozygous mutations in the genes encoding TGF-β receptors 1 and 2 (TGFBR1 and TGFBR2, respectively) cause Loeys-Dietz syndrome, an autosomal dominant aortic aneurysm syndrome, which predisposes patients to aggressive vascular disease with widespread systemic involvement (30). SMAD3 mutations play an important role in familial aortic diseases, characterized by aneurysms (31). Thus, *LDLRAD4* variants might affect AAD through the TGF-β–SMAD3 signaling axis in the general population.

The genetic basis of aortic dilation was initially explored through candidate gene approaches. Candidate genes were selected based on their biological relevance and their potential role in the pathogenesis of AAA. So far, genetic variants in 11 genes, including *SORT1, IL6R, LPA, AGTR1, TGFBR2, ACE* (Angiotensin I Converting Enzyme)*, MMP3, MMP13, MTHFD1* (Methylenetetrahydrofolate Dehydrogenase, Cyclohydrolase and Formyltetrahydrofolate Synthetase 1)*, MTRR* (5-Methyltetrahydrofolate-Homocysteine Methyltransferase Reductase) and *lrp5* (LDL Receptor Related Protein 5) mainly implicated in lipid metabolism, inflammation, blood pressure homeostasis, TGF-β signaling, degradation of extracellular matrix and methionine metabolism, have been subject of evaluation in candidate gene studies (15).

Furthermore, GWAS have so far identified genes in 15 chromosomal regions for AAA: *DAB2IP, LDLR, PSRC1-CELSR2-SORT1, CDKN2BAS1/ANRIL, IL6R, SMYD2, LINC00540, PCIF1-ZNF335-MMP9*, *ERG, AC012065.7/LDAH, MEPE, CDKN1A, RP11-136O12.2/TRIB1, LIPA, ZNF259/APOA5, ADAMTS8, CRISPLD2, CTAGE1, APOE, PCSK9, LPA, CHRNA3* and *ABHD16B* (10,16–21). We thus explored the potential role of these AAA-risk loci with aortic diameter. Only one of these risk loci, namely *CDKN2BAS1/ANRIL*, was associated with AAD in our combined meta-analysis. *ERG* gene was found to be associated at nominal significance. The lack of genetic overlap between both traits may be determined by the limited power of this study. Another explanation is that the disparities observed might indicate to what extend ageing and cellular senescence are important to form aneurysms. Genes like *IL6R, MMP9, MMP13* and *CDNK2* are intimately related to cellular senescence; hence cellular senescence-related pathways may have a great impact on the diameter of the abdominal aorta and its enlargement. *CDKN2BAS1/ANRIL*, located in the 9p21 chromosomic region, has been reported in numerous studies as a genetic risk locus for CAD, intracranial aneurysms and diverse cardiometabolic disorders (32).

From the gene-based meta-analysis of exome array data we identified an association between *PCSK5* and AAD. *PCSK5* encodes a member of the subtilisin-like proprotein convertase family, which is involved in the trafficking of peptide precursors through regulated or constitutive branches of the secretory pathway (33). This is also an important finding given that conditional inactivation of endothelial *PCSK5* has shown decreased collagen deposition in the heart and in the vasculature in aged mice, and may be relevant to aortic dilation biology (34). *PCSK5* is thought to process prorenin, pro-membrane type-1 matrix metalloproteinase as well as lipoprotein metabolism-related pathways (35,36). Furthermore, genetic variations at *PCSK5* locus have been associated with HDL-C levels possibly through the inactivation of endothelial lipase activity and atherosclerotic cardiovascular disease risk (36). *PCSK5* is also an important genetic predictor of tall stature as regulates the maturation of GDF15 (growth differentiation factor 15), a member of the TGF-β family, involved in body growth (37,38). Although evidence is scarce, the role of this gene on the pathology of aortic diameter and aortic enlargement might be through collagen regulation and inflammatory pathways modulated by cholesterol metabolism and/or via activation of the renin-angiotensin system (39). In light of our findings, further research may be warranted.

Through the implementation of Mendelian randomization methods, we examined and determined if AAA-related risk factors may be causally associated with the variation of infrarenal aortic diameter observed in the studied population. MR uses genetic variants as proxies for the risk factor and the outcome of interest and can offer an opportunity to shed light on the causality of risk factors-outcome associations (40). Unlike the associations of a risk factor with aortic enlargement reported from observational data, genetic associations are not influenced by reverse causation because the genotype is unmodified by the development of the disease. Moreover, the randomized assortment of parental alleles at conception tends to balance confounding factors among groups of differing genotypes (41).

In this study we used multiple independent SNPs as instrumental variables, selected from studies which have reported a robust association with each risk factor evaluated. We found evidence that genetic variation in height, PP and TG is associated with variation in AAD, consistent with causal associations. Height, among the body size measurements, has shown the strongest association with aortic size. Evidence has demonstrated that height-based relative aortic measure predicts the risk of rupture, dissection and death in patients with AAA (42). The association between height and AAA development and increasing risk of rupture might be explained by the presence of longer arteries in the AAA population (43). Nevertheless, the biological link between height and aortic enlargement remains unexplained.

Increased PP is associated with increased characteristic impedance (Zc) of the aorta and increased load on the heart, contributing to the risk of ischemia (44), diastolic dysfunction (45) and adverse clinical events (46,47). A high PP, markedly after mid-life when Zc increases rapidly, may have an impact on the aortic diameter (48). Evidence has shown that higher PP in older people is associated with smaller aortic lumen area and greater aortic wall stiffness and thickness (49). A small aortic diameter related with a higher PP suggests a mismatch in hemodynamic load accommodation by the heart and aorta in aged population. In contrast, a causal association between AAA and PP has not been established.

Pleiotropic effects are a major challenge in the implementation of Mendelian randomization (50). Instrumental variables that affect the outcome via pathways not including the exposure could distort or bias MR results. To address this, we performed sensitivity analyses including MR-Egger. Our MR-Egger result suggests a potential causal effect of TG on AAD. Prospective studies have shown positive relationships between TG and AAA (51–53). Moreover, TG is strongly related to risk of ruptured AAA (54). In line with our findings, a large meta-analysis MR study has reported significant associations between TG and AAA (55).

In contrast, we found that there was not significant evidence for other genetically determined lipid fractions such as HDL-C and LDL-C having a causal effect on AAD. One explanation is that the genetic variants for LDL-C and HDL-C may explain a small proportion of the total variance in these lipid traits and this could affect their association with aortic diameter. Another explanation is that both lipids are indeed not causally related to AAD.

Our findings support the clinical importance of the management of blood pressure/PP and TG for the prevention of AAA in high-risk patients. Therefore, targeting risk factors related with aortic expansion at early stages could have important implications for the implementation of public health interventions aiming to reduce the prevalence of these risk factors and the morbidity and mortality caused by AAA.

### Strengths and limitations

A key attribute of this study is the combination of a large discovery sample with standardized AAD measurement and dense 1000 Genomes imputation, which is the most common reference panel used in GWAS and offers excellent imputation for common variants (56). Differences in the methods employed to measure AAD [ultrasound, magnetic resonance imaging (MRI), computed tomography (CT)-scan] may have introduced site-based effects into the study conferring error or bias in the measurements, which ultimately may have contributed to a lack of associated variants observed. We made use of log-transformed AAD to standardize the phenotype and minimize the impact of the variability present among the cohorts. We acknowledge several limitations in our work. The sample size for this GWAS has been the main limitation that hampered identifying further loci. However, we have searched extensively to include all studies with relevant data. Sexual dimorphism is relevant as it has been observed in the development of AAA (57). However, we did not perform sex-specific analyses given our limited sample size. In the context of gene-based analysis of exome array data, simulation studies have shown that in certain scenarios SKAT-O (58,59) outperforms SKAT, as it combines both SKAT and burden tests, improving the power of the test. Therefore, we might have had a better statistical power if we had used SKAT-O. Our study population was of European ancestry. Generalizing the results to other ethnic groups should be done with caution.

## Conclusions

In summary, we identified one replicated locus and one suggestive locus associated with the diameter of infrarenal aortic aorta. In addition, we provided evidence that the main genetic determinants of PP and height also causally influence the diameter of the abdominal aorta. In contrast, we found that other genetically determined risk factors for vascular-related diseases had no effect on AAD. Our findings point to new biology as well as highlighting gene regions in mechanisms that have previously been implicated in the genetics of other vascular outcomes. Larger sample sizes together with functional studies, investigating the translational potential of these observations, are critical to characterize the molecular mechanisms regulated by the genes described in this study.

## Materials and Methods

### Participating studies

The study was done primarily in the framework of the Cohorts for Heart and Aging Research in Genomic Epidemiology (CHARGE) Consortium, an international collaborative effort to facilitate GWAS meta-analysis and replication opportunities among multiple large cohorts (60). The discovery panel included European American participants from the Cardiovascular Health Study_European (CHS) (GWAS, *n* = 2699; exome array, *n* = 3294), Cooperative Health Research in the Region Augsburg (KORA) (GWAS, *n* = 354; exome array, *n* = 337), Framingham Heart Study_European (FHS) (GWAS and exome array, *n* = 3262), Study of Health in Pomerania (SHIP-2 and SHIP-T) [GWAS, *n* = 1010 (SHIP-2) and 827 (SHIP-Trend); exome array, *n* = 2848], The Multi-Ethnic Study of Atherosclerosis_European (MESA) (GWAS, *n* = 750; exome array, *n* = 740) and the RS (GWAS, *n* = 3913; exome array, *n* = 327) and BIOIMAGE (exome array, *n* = 3672). As a replication study, we used the PBIO1 cohort (GWAS, *n* = 727). Details of the cohorts included are provided in supplementary information (Supplementary Material, Tables S1a, b and S2).

### Abdominal aortic phenotypes

Maximum aortic diameter was defined as the largest measurement of anterior–posterior diameter of the infrarenal aorta measured by MRI, ultrasound or CT-scan using previously described reading protocols. Due to positive skewness, we used natural log-transformed AAD measurements. A description of the method employed in AAD measurement by each study is provided in methods in the supplementary information.

### Genotyping and imputation

The studies employed SNP arrays available from Illumina or Affymetrix. Using available imputation techniques, each cohort imputed approximately > 37 million variants from 1000 Genomes reference panel (phase 1 version 3) and applied strict quality control checks. Further information on the genotyping and imputation methods is detailed in Supplementary Material, Table S3**.**

Exonic and non-exonic variants were genotyped using the Illumina Infinium HumanExome BeadChip kit. The array covers > 240 000 markers, mostly coding variants discovered through exome sequencing in approximately 12 000 individuals and observed at least three times across at least two existing sequence datasets, and includes nonsynonymous, splicing, stop-altering variants, most of which are rare (http://genome.sph.umich.edu/wiki/Exome_Chip_Design). In order to ensure the accurate identification of variants and to minimize population stratification, exome array data quality control was performed based on Best Practices and Joint Calling of the HumanExome BeadChip: The CHARGE Consortium (61). Further details on methods employed by each study are outlined in supplementary information.

### Statistical analyses within studies

Each study implemented GWAS and exome array data analyses based on a predefined analysis plan. We conducted linear regression models to evaluate the association of log-transformed AAD with genetic variations. For each variant, each study fitted additive genetic models regressing trait on genotype dosage (0–2 copies of the variant allele). In the primary model, each regression was adjusted by sex, age and principal components. Further adjustments including study site and/or familial structure were performed if necessary. We determined the association of each SNP with natural logarithm transformed AAD as the regression slope, its standard error and its corresponding *P*-value. Furthermore, we examined 23 SNPs previously identified for AAA in GWAS and sought replication in our study (10,16–18,21,62).

To conduct study-specific analyses on exome array data, all the studies used the prepScores function, implemented in the R package ‘SeqMeta’ (63). Briefly, the prepScores function calculates scores and MAF for each variant interrogated within a gene. Log-transformed AAD was regressed against sex, gender and principal component analysis. Subsequently, the calculated prepScores by each cohort were combined in a single variant meta-analysis and gene-based meta-analysis.

### Genome-wide association study quality control and meta-analysis

Each cohort conducted a GWAS on AAD in adult subjects with genetic information available. Subsequently, GWAS summary statistics were uploaded to a central repository for QC and meta-analysis. We conducted data quality control at study file level of GWAS outputs using EasyQC (64) to identify file naming errors, erroneous SNP genotype data and false association caused by incorrect analysis models. We filtered out variants with a poor imputation quality (SNPTEST INFO value or MACH RSQR < 0.4, Probabel < 0.3) prior quality control. We further calculated genomic inflation for each study to determine population stratification or any other inconsistencies that might have inflated the test statistics. After QC, we meta-analyzed a range of 8.8–11.1 million of variants per study. The meta-analysis of linear regression estimates and standard errors using an inverse-variance weighting approach was conducted using METAL (65). We reported SNPs that were present in at least five studies and with an average MAF of at least 1%. Variants with a meta-analyzed *P*-value ≤ 5 × 10^−8^ were considered significant.

A gene-set analysis of GWAS data was done using MAGMA v1.06 (66). Gene-based results were used to perform a tissue enrichment analysis implemented in Genotype-Tissue Expression Project (GTEx). Additional information is provided in supplementary information.

### Meta-analysis of rare exonic variants from exome array

Study-specific summary statistics, such as estimated regression coefficient for each variant and its standard error (prepScores) were meta-analyzed on single variant level, to perform score tests for single SNP associations, and in a gene-based test [sequence kernel association test (SKAT)] using seqMeta (http://cran.r-project.org/web/packages/seqMeta/index.html) (63). SKAT directly performs multiple regressions models, neither directionality nor magnitudes of the associations are assumed a priori but are instead estimated from the data (67). Hence, SKAT is robust to the magnitude and direction of genetic effects as well as to the presence of neutral variants (67). Single variant results were filtered for a pooled MAF > 0.005 and those included in at least 50% of the cohorts were reported. Furthermore, variants with MAF ≤ 5% were included in the gene-level test. Additional arguments implemented in SKAT meta-analysis allowed us to specify the method of *P*-values calculation. We used the default method ‘saddlepoint,’ which appears to have higher relative accuracy (63,68). We defined a statistical significance threshold of single variant and gene-based exome array meta-analysis based on Bonferroni correction for multiple testing, ~55 434 variants (*P*-value < 9 × 10^−7^) and ~16 378 genes (*P*-value < 3 × 10^−6^), respectively. Genetic variants in exome array were annotated using SNPInfo file 12 version 1.0.

### SNP heritability and genetic correlation

To characterize the extent to which SNPs determine AAD, and shared genetic etiology with other traits (AAA, coronary artery disease and stroke), we applied linkage disequilibrium score regression (LDSC) (69). We used the default European LD score file based on the European 1KG reference panel. The analyses were conducted using LDSC (LD SCore) v1.0.1. (69,70). Further information is detailed in supplementary information.

### Identification of expression quantitative trait loci

Furthermore, we examined the effect of associated genetic variants on the expression of genes in *cis* and *trans.* To characterize their effects, we first sought SNP associated with gene expression from the GTEx (GTEx portal, Analysis Release V7) a platform with available expression data on potential target organs (heart tissue, kidney tissue, brain tissue, aortic endothelial cells and blood vessels) as well as blood cell types (CD4+ macrophages, monocytes) (71). The gene expression values are shown in TPM, calculated from a gene model with isoforms collapsed to a single gene. Box plots are shown as median and 25th and 75th percentiles, and outliers are displayed as dots if they are above or below 1.5 times the interquartile range (71). In addition, the platform also allows the assessment of isoform expression generated using RSEM, software package for estimating gene and isoform expression levels from RNA-Seq data.

### Genetic risk score and Mendelian randomization analysis

In order to determine the additive association of AAA-related SNPs on AAD, we combined the effect size of each AAA-SNP reported up to date in a weighted genetic risk score, and tested its association with AAD in the RS Cohort (*n* = 3913).

We implemented a two-sample Mendelian randomization analysis to evaluate causal effects of a number of risk factors on AAD. Risk factors were chosen based on previous literature reporting them to be associated with AAA. We identified genetic instruments for SBP (72,73), DBP (72,73), PP (72,73), smoking (74,75), LDL, HDL and TG (76,77), height (78) and BMI (79,80) using the most up-to-date GWAS on these traits. We used the IVW method to combine the effect of multiple instruments. We further used two sensitivity analyses, the weighted median and MR-Egger, to investigate potential effect of pleiotropic variants on the estimates. For lipid traits, we used a multivariable MR method (81). In this approach, a single regression model with outcome variable (β for AAD) was fitted for each of the predictor variables (β for LDL, β for HDL and β for TG). The model was implemented as a multilinear regression of SNP association estimates weighted by the inverse variances of the estimated associations of SNPs with the outcome.

All MR methods for multiple genetic instruments were conducted using ‘MendelianRandomization,’ a statistical package running under R (https://cran.r-project.org/web/packages/MendelianRandomization/index.html) (82). We used MR-PRESSO (Mendelian randomization pleiotropy residual sum and outlier) to identify horizontal pleiotropic outliers in multi-instrument summary-level MR testing (https://github.com/rondolab/MR-PRESSO) (83). Further details are outlined in supplementary information.

## Supplementary Material

4_Supplementary_tables_S1_S14_ddac051

3_Supplementary_information_for_resubmission2ndRev_ddac051
