# Supplementary material for: Genetic and clinical determinants of abdominal aortic diameter: genome-wide association studies, exome array data and Mendelian randomization study
Source: Hum Mol Genet. 2022 Mar 2;31(20):3566–79. doi: 10.1093/hmg/ddac051 (PMC9558840; doi:10.1093/hmg/ddac051)
Supplement: 3_Supplementary_information_for_resubmission2ndRev_ddac051 [file 3_supplementary_information_for_resubmission2ndrev_ddac051.docx]

**Supplementary Information**

**Genetic and clinical determinants of abdominal aortic diameter: Genome-wide association studies, exome array data and Mendelian randomization study**

**Table of Content**

1. **Supplemental Methods:**
   1. Study-specific methods section.
   2. Genetic risk score and Mendelian randomization analyses
   3. Gene set enrichment analysis
   4. SNP heritability and genetic correlation
2. **Supplemental Figures:**
   1. Figure S1. Forest plot for the association of rs74448815 with abdominal aortic diameter in different cohorts
   2. Figure S2. Overall gene expression of *LDLRAD4* across several tissues
   3. Figure S3. Quantile-quantile (Q-Q) plot gene-based exome array analysis
   4. Figure S4. Mendelian Randomization plot height SNPs and AAD
   5. Figure S5. Mendelian Randomization plot pulse pressure SNPs and AAD
   6. Figure S6. Mendelian Randomization plot LDL SNPs and AAD
   7. Figure S7. Mendelian Randomization plot HDL SNPs and AAD
   8. Figure S8. Mendelian Randomization plot triglycerides SNPs and AAD
   9. Figure S9. Mendelian Randomization plot systolic blood pressure SNPs and AAD
   10. Figure S10. Mendelian Randomization plot diastolic blood pressure SNPs and AAD
   11. Figure S11. Mendelian Randomization plot smoking SNPs and AAD
   12. Figure S12. Mendelian Randomization plot BMI SNPs and AAD
3. **Funding and acknowledgements**

**1. Supplemental Methods**

**a. Study-specific methods section**

**Cardiovascular Health Study**

*Cohort summary*

The Cardiovascular Health Study (CHS) is a population-based cohort study of risk factors for coronary heart disease and stroke in adults ≥65 years conducted across four field centers (1) . The original cohort of 5,201 persons, predominantly of European ancestry, was recruited in 1989-1990 from random samples of the Medicare eligibility lists; subsequently, an additional predominantly African-American cohort of 687 persons was enrolled in 1992-1993 for a total sample of 5,888.

*Abdominal aortic diameter measurement*

Ultrasonographers who were trained at a central location obtained ultrasonographic B-mode gray-scale images of the abdominal aorta in transverse and longitudinal projections. The suprarenal measure of aortic diameter was taken 1 cm distal to the origin of the superior mesenteric artery, just above the level of the left renal artery. Either the superior mesenteric artery or the renal artery had to be visualized to accept the image for measurement. The infrarenal measure of aortic diameter was determined by the site of the maximum diameter aortic artery (lumen plus wall) below the renal arteries.

**Cooperative Health Research in the Region Augsburg (KORA)**

*Cohort summary*

The KORA MRI substudy is a case control study to investigate differences in subclinical disease between diabetic, prediabetic and normoglycemic participants. The substudy is nested in the second follow-up (KORA FF4, enrollment 2013/2014) of the KORA S4 study. KORA S4 is a prospective survey from the general population consisting of residents in the region of Augsburg aged 25–74 years recruited between 1999 and 2001. For KORA MRI, eligible subjects were selected if they met the following inclusion criteria: willingness to undergo whole-body MRI and qualification in either the prediabetes, diabetes, or control group. The following exclusion criteria were applied: Age > 72 years, subjects with validated/self-reported stroke, myocardial infarction or revascularization, cardiac pacemaker or implantable defibrillator, cerebral aneurysm clip, neural stimulator, any type of ear implant, ocular foreign body (e.g. metal shavings), any implanted device (e.g. insulin pump, drug infusion device), pregnant or breast feeding female subjects, or subjects with claustrophobia, known allergy against gadolinium compounds, or serum creatinine =1.3 mg/dL (2).

*Abdominal aortic diameter measurement*

Whole-body MRI was performed at a 3 Tesla Magnetom Skyra (Siemens AG, Healthcare Sector, Erlangen Germany). Images from the gradient echo Dixon sequence were used to determine the maximum diameter of the aorta abdominalis. The 3D function syngo (Siemens) was used to localize the aorta infrarenalis in the coronal and sagittal plane. The anterior-posterior diameter of the aorta infrarenalis was determined manually in strictly axial direction.

**Rotterdam Study**

*Cohort summary*

The Rotterdam Study (RS) is a prospective study, population-based cohort study ongoing since 1990 including population from the well-defined Ommoord district in the city of Rotterdam. Initially, the study included 7,983 individuals 55 years aged or older. In 2000, 3,011 additional participants who had become 55 years or moved into the study district were included to the cohort. In 2006, the RSIII cohort was established including 3,932 subjects aged 45-54 years. As of 2008, the Rotterdam Study cohort comprises a total of 14,926 subjects aged 45 years. All individuals comprised in this study were of European and African descent. The study has conducted extensive clinical examinations, repeated every 3–4 years, to investigate the causes and risk factors associated with cardiovascular diseases, cognitive-related disorders, respiratory diseases, liver diseases, diabetes mellitus, cancer, among others (3).

*Abdominal aortic diameter measurement*

Abdominal aortic diameter was measured in the infrarenal portion of the abdominal aorta using Ultrasound. The subject was examined in supine position, flexion of the hips and knees by putting a pillow under the subject’s knees. The transducer was placed in midline of the abdomen, perpendicular to the axis of the abdominal aorta. The longitudinal identification of the aorta was done from xiphoid process to umbilicus. The scanning was performed from proximal of superior mesenteric artery in distal direction. Maximum aortic diameter was measured in antero-posterior direction, around 1 cm distal from origin of the superior mesenteric artery, just above the level of the left renal artery (“proximal artery”). All measurements were done including both vessel walls.

**Study of health in Pomerania (SHIP-Trend and SHIP-2)**

*Cohort summary*

The Study of Health In Pomerania is a prospective longitudinal population-based cohort study in Western Pomerania assessing the prevalence and incidence of common diseases and their risk factors. SHIP encompasses two independent cohorts: SHIP and SHIP-TREND. Participants aged 20 to 79 with German citizenship and principal residency in the study area were recruited from a random sample of residents living in the three local cities, 12 towns as well as 17 randomly selected smaller towns. Individuals were randomly selected stratified by age and sex in proportion to population size of the city, town or small towns, respectively. A total of 4,308 participants were recruited between 1997 and 2001 in the SHIP cohort. Between 2008 and 2012 a total of 4,420 participants were recruited in the SHIP-TREND cohort. Individuals were invited to the SHIP study centre for a computer-assisted personal interviews and extensive physical examinations (4).

*Abdominal aortic diameter measurement*

MRI was performed with a 1.5-T MRI scanner (Magnetom Avanto; Siemens Healthcare, Erlangen, Germany) using integrated coil elements and phased-array surface coils. Aortic diameters were measured in a 3-Dimensional T1-weighted volumetric interpolated breath-hold examination (VIBE) sequence, with an axial orientation acquired with 4.0 mm slice thickness and the following imaging parameters; repetition time TR: 7.5 ms; echo time TE: 2.4 ms; flip angle FA: 10°; pixel size 2.4x1.6 mm, with a gap size of 0.3 mmand a bandwith of 285 Hz/Px. The maximum outer diameter of the infrarenal aorta abdominalis (defined as 1 cm above/below the right renal artery origin) were analyzed using the OsiriX image viewing and processing software (version 3.6.1; Pixmeo Sarl, Bernex, Switzerland).

**Framingham Heart Study (FHS)**

*Cohort summary*

Framingham Heart Study (FHS) is a community-based prospective study designed to investigate the incidence of cardiovascular diseases and related risk factors. FHS population include family members of three generations. Participants of the first generation was recruited in 1948 with bi-annual follow-up examination. Children and their spouse of the first generation participants were invited for the offspring study beginning at 1971 with follow-up examinations of every four years. In 2005, children of the offspring cohort were invited to start the third generation study with a follow-up examination of four years. A multi-detector computed tomography (MDCT) sub-study I (2002-2005) was conducted in a total of 1,200 offspring, and 2,200 of generation 3 participants had consented to participated in the MDCT study. Information on clinical characteristics for the offspring participants, those who had attended their 7th examination cycle and the first examination cycle for the third generation participants, was abstracted to merge with CT results for analysis (5-7).

*Abdominal aortic diameter measurement*

Diameters of the main abdominal aorta were obtained at locations identified by anatomical landmarks using the CT scan films.

**Multi-ethnic Study of Atherosclerosis**

*Cohort summary*

The Multi-Ethnic Study of Atherosclerosis (MESA) was initiated in July 2000 to investigate the prevalence, correlation, and progression of subclinical cardiovascular disease in a population-based sample of 6,814 ethnically diverse men and women aged 45-84 years. Approximately 38% of the cohort are Caucasian, 28% African-American, 23% Hispanic, and 11% Asian (of Chinese descent). The cohort was recruited from six Field Centers in the United States and characterized with respect to coronary calcification using computed tomography, ventricular mass and function using magnetic resonance imaging, and other measures at baseline (8) .

*Abdominal aortic diameter measurement*

Participants underwent CT scanning of the chest and abdomen to ascertain the presence and extent of coronary artery calcium and abdominal aorta calcium. Images from the abdominal CT scans were retrospectively interrogated to determine the diameter of the abdominal aorta using computer software (Osiris 4.19, University of Geneva, Geneva, Switzerland). Measures were conducted at 5 cm proximal to the aortic bifurcation and at the slice just above the aortic bifurcation. An adjustable-size electronic caliper in the shape of a circle was used to measure the diameter (d) by fitting the caliper around the circumference (C) of the adventitia of the aorta. The computer then calculated the diameter from the circumference measurement using the equation, d Z C/p. Each location was measured three times by a single reader who was unaware of subject characteristics; the average of these measurements was used in the analysis; the intraclass correlation was 0.93, average difference between measurements was 4.0%.

**PBIO**

*Cohort summary*

The Partners Healthcare Biobank is a large research data and sample repository working within the framework of Partners Personalized Medicine (9). It provides researchers access to high quality, consented samples to help foster research, advance understanding of the causes of common diseases, and advance the practice of medicine. The Partners Biobank provides banked samples (plasma, serum and DNA) collected from consented patients. These samples are available for distribution to Partners HealthCare investigators with appropriate approval from the Partners Institutional Review board (IRB). They are linked to clinical data that originates in the Electronic Medical Record (EMR), as well as additional health information collected in a self-reported survey.

*Abdominal aortic diameter measurement*

Abdominal aortic diameter was measured in the infrarenal portion of the abdominal aorta using MRI or CT scan. The subject was examined in supine position and maximum aortic diameter was measured in antero-posterior direction. All measurements were done including both vessel walls.

**BIOIMAGE**

*Cohort summary*

The BioImage study (NCT00738725) is a multi-ethnic, observational study aimed at characterizing subclinical atherosclerosis in 6,699 US adults (55-80 years at baseline, 2008- 2009) at risk for, but without, clinical atherosclerotic cardiovascular disease. The study is

designed to evaluate associations among imaging and circulating biomarkers (cross-sectional) and their ability to predict atherothrombotic events (longitudinal) in asymptomatic at-risk subjects (10).

*Abdominal aortic diameter measurement*

Abdominal aorta was initially assessed by ultrasound. Participants with one or more abnormal screening test results underwent advanced imaging with contrast-enhanced magnetic resonance imaging for carotid and aortic plaques (10).

**Million Veteran Program**

*Cohort summary*

The Million Veteran Program (MVP) was established in 2011 as a national research initiative to determine how genetic variation influences the health of US military veterans. In the MVP, individuals aged 19 to over 100 years were recruited from 63 Veterans Affairs Medical Centers across the USA. In this analysis, we performed a genome-wide association study in the Million Veteran Program testing ≈18 million DNA sequence variants with AAA (7642 cases and 172 172 controls) in veterans of European ancestry

*Definition of cases with abdominal aortic aneurysm*

Individuals were defined as having abdominal aortic aneurysm (AAA) or being a disease-free control using a previously adjudicated[13](https://www.ahajournals.org/reader/content/179cac0a396/10.1161/CIRCULATIONAHA.120.047544/format/epub/EPUB/xhtml/index.xhtml#R13) definition initially proposed by Denny et al(11) .[14](https://www.ahajournals.org/reader/content/179cac0a396/10.1161/CIRCULATIONAHA.120.047544/format/epub/EPUB/xhtml/index.xhtml#R14) AAA cases were defined as the presence of 2 instances of any of the following International Classification of Diseases (ICD)–9 or ICD-10 codes in a participant’s EHR: 441.3, 441.4, I71.3, or I71.4. Controls were defined as possessing no occurrences of the aforementioned ICD codes, as well as no occurrences of the ICD-9 codes 440 through 448 or ICD-10 codes I71 through I75, I77 through I79, or K55.

**b. Genetic risk score and Mendelian randomization analysis**

In order to determine the additive association of AAA-related SNPs on AAD, we combined the effect size of each AAA-SNP reported up to date in a weighted genetic risk score (GRS). The GRS was constructed for AAD by multiplying the number of effect alleles at each AAA-associated locus by the corresponding reported β coefficient from the GWAS and then summing the products. The total score was then divided by the average effect size multiplied by 100 to rescale the scores and standardize them to a range between 0 and 100. Subsequently, the GRS was used as a predictor for AAD in a linear regression model adjusted by age, sex and cohort. The analyses were done in 3,913 subjects from Rotterdam Study.

We implemented a Mendelian randomization analysis to establish the causal association of AAA-related risk factors on abdominal aortic diameter. We selected genetic instruments using the largest GWAS on blood pressure, smoking, low-density lipoprotein (LDL), height and body mass index (BMI). We examined 104 SNPs reported for SBP (12, 13), 139 SNPs associated with DBP (12, 13), 109 SNPs reported for pulse pressure (12, 13), 68 variants associated with smoking (14, 15), 66 SNPs for LDL (16, 17), 39 SNPs for HDL (16, 17), 35 SNPs for triglycerides (16, 17), 134 SNPs associated with height (18) and 73 SNPs found for BMI (19, 20). For lipid traits, we used both a multivariable and conventional MR methods (21). In the multivariable MR, a single regression model with outcome variable (β for AAD) was fitted for each of the predictor variables (β for LDL, β for HDL, and β for TG). The model was implemented as a multilinear regression of SNP association estimates weighted by the inverse variances of the estimated associations of SNPs with the outcome. For the multivariable MR on overall lipid traits we used 66 SNPs.

Conventional Mendelian randomization analyses were performed by implementing inverse-variance weighted (IVW)- a linear regression of the SNP-risk factors estimates on SNP-abdominal aortic diameter estimates-. In IVW, the overall causal estimate assumes that the ratio estimates for each instruments is independent of others (22). Therefore, correlated SNPs were pruned (LD cutoff=0.3) and independent SNPs were used in the analyses. SNP pruning was performed using R package ‘SNPRelate’ (23). We established statistical significance using a two-sided alpha of 0.05. Sensitivity analyses, using penalized weighted median Mendelian randomization and MR-Egger regression, were implemented to investigate the potential presence of unbalanced horizontal pleiotropy among the genetic instruments. Weighted median method gives more weight to genetic variants close to the median causal estimate, and yields robust results even when up to 50% of the information comes from invalid instrumental variables (24). MR-Egger regression was applied as described by Bowden et al (25). MR-Egger method is able to assess whether genetic variants have pleiotropic effects on the outcome that differ on average from zero (directional pleiotropy), and provides a causal estimate even if all the genetic variants present pleiotropic effects (as per the InSIDE rule) (25). The conventional and sensitivity analyses of MR were conducted using “MendelianRandomization”, a statistical package running under R (26) (<https://cran.r-project.org/web/packages/MendelianRandomization/index.html>).

1. **Gene set enrichment analysis**

A gene-set analysis of GWAS data was done using MAGMA v1.06 (27), implemented by FUMA v1.3.2.(28). Gene-based approach aims to assess the joint association of multiple SNPs within a gene with abdominal aortic diameter. Gene-set analysis aggregates individual genes to groups of genes sharing certain biological, or other functional characteristics, allowing the identification of effects consisting of multiple weaker associations to determine their joint effect. Likewise, gene-set analysis might provide insight into the involvement of specific biological pathways or cellular functions underlying AAD. (27) The analysis was performed using summary-level meta-analysis results. For all MAGMA analyses multiple testing was accounted for Bonferroni correction.

1. **SNP heritability and genetic correlation**

To characterize the extent to which common genetic variants determine AAD, and shared genetic etiology with other traits (AAA, coronary artery disease and stroke), we applied linkage disequilibrium score regression (LDSC) (29) methods for estimating SNP heritability and genetic correlation based on genome-wide sharing between distantly related individuals. LDSC is a summary-statistics-based method which estimates heritability and genetic correlation while accounting for LD and requires only publicly available summary statistics from genetic studies.(30) In brief, the cross-product of two GWAS test statistics is calculated at each genetic variant, and this cross-product is regressed on the LD score. The slope of the regression is used for estimating the genetic covariance between two phenotypes. We used the default European LD score file based on the European 1KG reference panel. The summary statistics of GWAS on AAA was obtained from Klarin et al (31); genetic association data on coronary artery disease was obtained from van der Harst et al (32), and the summary association statistics for stroke published by Malik et al (33) was used. The analyses were conducted using LDSC (LD SCore) v1.0.1 package running under R (<https://github.com/bulik/ldsc>) (29, 34).

1. **Supplemental Figures:**
2. Figure S1. Forest plot for the association of rs74448815 with abdominal aortic diameter in different cohorts.





Study specific estimates and summary association between rs74448815 and abdominal aortic diameter are shown.

1. Figure S2. Overall gene expression of *LDLRAD4* across several tissues


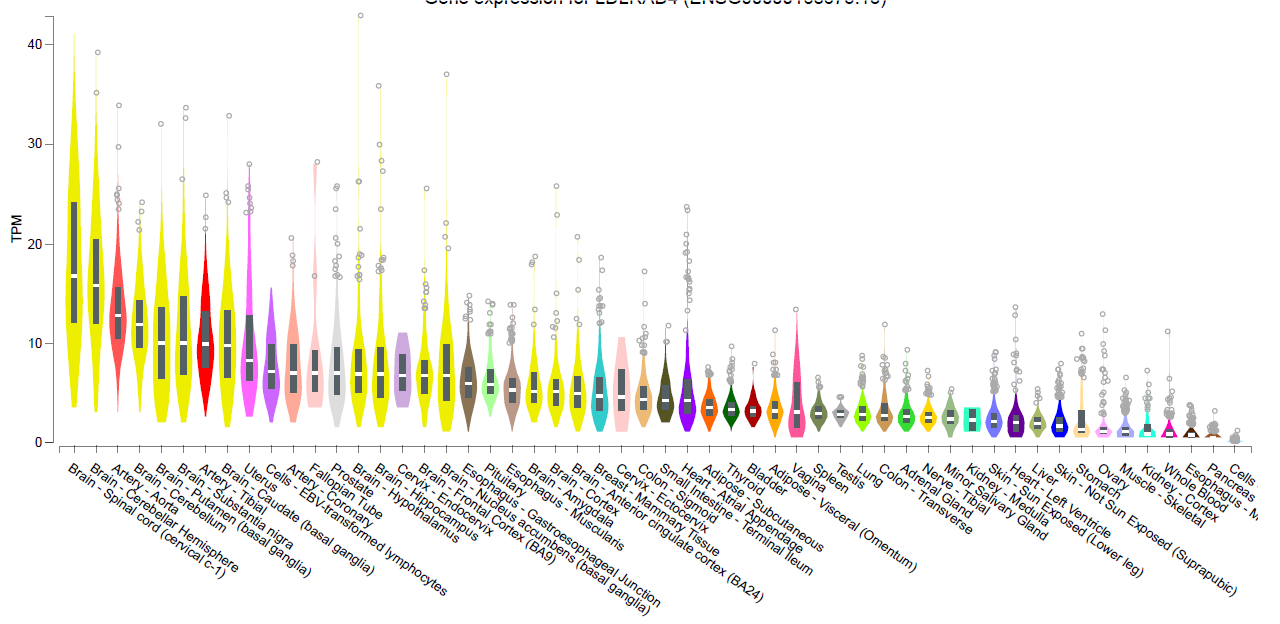


Violin plot depicting expression quantitative trait loci associated to *LDLRAD4.* Gene and transcript expression on the GTEx Portal are shown in Transcripts Per Million (TPM) and measured in 49 tissues. The values are linear scaled and sorted by the median. Each ‘violin’ represents the TPM estimates per tissue and the shape represents the density estimate of the expression levels. This Figure is downloaded from the official GTEx (https://www.gtexportal.org). GTEx=Genotype-Tissue Expression.

1. Figure S3. Quantile-quantile (Q-Q) plot gene-based exome array analysis


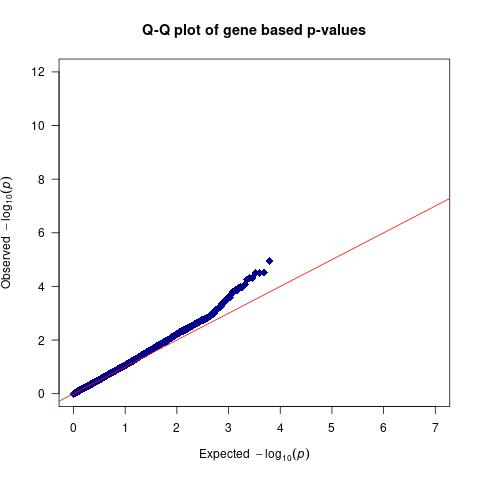


1. Figure S4. Mendelian Randomization plot height SNPs and AAD





1. Figure S5. Mendelian Randomization plot pulse pressure SNPs and AAD





1. Figure S6. Mendelian Randomization plot LDL SNPs and AAD





1. Figure S7. Mendelian Randomization plot HDL SNPs and AAD





1. Figure S8. Mendelian Randomization plot triglycerides SNPs and AAD





1. Figure S9. Mendelian Randomization plot systolic blood pressure SNPs and AAD





1. Figure S10. Mendelian Randomization plot diastolic blood pressure SNPs and AAD





1. Figure S11. Mendelian Randomization plot smoking SNPs and AAD





1. Figure S12. Mendelian Randomization plot BMI SNPs and AAD





1. **Funding and acknowledgements**

**Cardiovascular Health Study**

This CHS research was supported by NHLBI contracts HHSN268201200036C, HHSN268200800007C, HHSN268201800001C, N01HC55222, N01HC85079, N01HC85080, N01HC85081, N01HC85082, N01HC85083, N01HC85086; and NHLBI grants U01HL080295, R01HL087652, R01HL105756, R01HL103612, R01HL120393, and U01HL130114 with additional contribution from the National Institute of Neurological Disorders and Stroke (NINDS). Additional support was provided through R01AG023629 from the National Institute on Aging (NIA). The provision of genotyping data was supported in part by the National Center for Advancing Translational Sciences, CTSI grant UL1TR001881, and the National Institute of Diabetes and Digestive and Kidney Disease Diabetes Research Center (DRC) grant DK063491 to the Southern California Diabetes Endocrinology Research Center. A full list of principal CHS investigators and institutions can be found at CHS-NHLBI.org. The content is solely the responsibility of the authors and does not necessarily represent the official views of the National Institutes of Health.

**Cooperative Health Research in the Region Augsburg (KORA)**

The KORA study was initiated and financed by the Helmholtz Zentrum München – German Research Center for Environmental Health, which is funded by the German Federal Ministry of Education and Research (BMBF) and by the State of Bavaria. Furthermore, KORA research was supported within the Munich Center of Health Sciences (MC-Health), Ludwig-Maximilians-Universität, as part of LMUinnovativ.

**Rotterdam Study**

This research was primarily supported by the Erasmus MC and Erasmus University Rotterdam; the Netherlands Organization for Scientific Research (NWO); the Netherlands Organization for Health Research and Development (ZonMw); the Research Institute for Diseases in the Elderly (RIDE); the Netherlands Genomics Initiative (NGI); the Ministry of Education, Culture and Science, the Ministry of Health, Welfare and Sports; the European Commission (DG XII); and the Municipality of Rotterdam. This work was also supported by Dutch Heart Foundation [grant number 2015T094 (to A.J.M.R)]. The contribution of the inhabitants, general practitioners, and pharmacists of the Ommord district to the Rotterdam Study is gratefully acknowledge.

**Study of health in Pomerania (SHIP-Trend and SHIP-2)**

SHIP is part of the Community Medicine Research Network of the University Medicine Greifswald, which is supported by the German Federal State of Mecklenburg- West Pomerania.

**Framingham Heart Study (FHS)**

The Framingham Heart Study laboratory work for this project was funded by National Institutes of Health contract N01-HC-25195. The analytical component of this project was funded by the Division of Intramural Research, National Heart, Lung, and Blood Institute, National Institutes of Health, Bethesda. The views expressed in this manuscript are those of the authors and do not necessarily represent the views of the National Heart, Lung, and Blood Institute; the National Institutes of Health; or the U.S. Department of Health and Human Services.

**Multi-ethnic Study of Atherosclerosis**

MESA and the MESA SHARe project are conducted and supported by the National Heart, Lung, and Blood Institute (NHLBI) in collaboration with MESA investigators. Support for MESA is provided by contracts 75N92020D00001, HHSN268201500003I, N01-HC-95159, 75N92020D00005, N01-HC-95160, 75N92020D00002, N01-HC-95161, 75N92020D00003, N01-HC-95162, 75N92020D00006, N01-HC-95163, 75N92020D00004, N01-HC-95164, 75N92020D00007, N01-HC-95165, N01-HC-95166, N01-HC-95167, N01-HC-95168, N01-HC-95169, UL1-TR-000040, UL1-TR-001079, UL1-TR-001420. Also supported in part by the National Center for Advancing Translational Sciences, CTSI grant UL1TR001881, and the National Institute of Diabetes and Digestive and Kidney Disease Diabetes Research Center (DRC) grant DK063491 to the Southern California Diabetes Endocrinology Research Center.

SHARe genotyping and Exome Chip genotyping: Funding for SHARe genotyping was provided by NHLBI Contract N02-HL-64278. Genotyping was performed at Affymetrix (Santa Clara, California, USA) and the Broad Institute of Harvard and MIT (Boston, Massachusetts, USA) using the Affymetrix Genome-Wide Human SNP Array 6.0. Provision of exome chip genotyping was provided in part by support of NHLBI contract N02-HL-64278 and UCLA CTSI UL1-TR001881, and the S.Calif DRC DK063491.

**PBIO**

The Partners Biobank leaders wish to thank the many participants in the Partners HealthCare Biobank for their willingness to engage in a research program to advance our understanding of human health. Grant support: 1 U01 HG008685-01, from the National Institute for Human Genome Research.

**BIOIMAGE**

The High Risk Plaque (HRP) Initiative encompassing the BioImage Study is a precompetitive industry collaboration funded by Abbott, Abbvie, AstraZeneca, BG Medicine, Merck, Philips, and Takeda. HRP Joint Steering Committee: Pieter Muntendam, MD (BG Medicine); Aram Adourian (BG Medicine); Michael Klimas, PhD (Merck); Joel Raichlen, MD (AstraZeneca); Oliver Steinbach (Philips); James Beckett (Philips); Ramon Espaillot (Abbvie); Michael Jarvis (Abbvie) and Tomoyuki Nishimoto (Takeda). The sponsor had no role in the study design; in the collection, analysis, and interpretation of the data; in the writing of this report; or in the decision to submit the paper for publication.

**Million Veteran Program**

This research is based on data from the Million Veteran Program, Office of Research and Development, Veterans Health Administration. This publication does not represent the views of the Department of Veteran Affairs or the United States Government.

**References**

1. Fried LP, Borhani NO, Enright P, Furberg CD, Gardin JM, Kronmal RA, et al. The Cardiovascular Health Study: design and rationale. Ann Epidemiol. 1991;1(3):263-76.

2. Bamberg F, Hetterich H, Rospleszcz S, Lorbeer R, Auweter SD, Schlett CL, et al. Subclinical disease burden as assessed by whole-body MRI in subjects with prediabetes, subjects with diabetes, and normal control subjects from the general population: the KORA-MRI study. Diabetes. 2017;66(1):158-69.

3. Hofman A, Brusselle GG, Murad SD, van Duijn CM, Franco OH, Goedegebure A, et al. The Rotterdam Study: 2016 objectives and design update. European journal of epidemiology. 2015;30(8):661-708.

4. Volzke H, Alte D, Schmidt CO, Radke D, Lorbeer R, Friedrich N, et al. Cohort profile: the study of health in Pomerania. Int J Epidemiol. 2011;40(2):294-307.

5. Dawber TR, Kannel WB. The Framingham Study an epidemiological approach to coronary heart disease. Circulation. 1966;34(4):553-5.

6. Feinleib M, Kannel WB, Garrison RJ, McNamara PM, Castelli WP. The Framingham offspring study. Design and preliminary data. Preventive medicine. 1975;4(4):518-25.

7. Hoffmann U, Massaro JM, Fox CS, Manders E, O'Donnell CJ. Defining normal distributions of coronary artery calcium in women and men (from the Framingham Heart Study). Am J Cardiol. 2008;102(9):1136-41, 41 e1.

8. Bild DE, Bluemke DA, Burke GL, Detrano R, Diez Roux AV, Folsom AR, et al. Multi-ethnic study of atherosclerosis: objectives and design. American journal of epidemiology. 2002;156(9):871-81.

9. Karlson E, Boutin N, Hoffnagle A, Allen N. Building the partners healthcare biobank at partners personalized medicine: informed consent, return of research results, recruitment lessons and operational considerations. Journal of personalized medicine. 2016;6(1):2.

10. Muntendam P, McCall C, Sanz J, Falk E, Fuster V, High-Risk Plaque I. The BioImage Study: novel approaches to risk assessment in the primary prevention of atherosclerotic cardiovascular disease—study design and objectives. American heart journal. 2010;160(1):49-57. e1.

11. Denny JC, Bastarache L, Ritchie MD, Carroll RJ, Zink R, Mosley JD, et al. Systematic comparison of phenome-wide association study of electronic medical record data and genome-wide association study data. Nature biotechnology. 2013;31(12):1102-11.

12. Evangelou E, Warren HR, Mosen-Ansorena D, Mifsud B, Pazoki R, Gao H, et al. Genetic analysis of over 1 million people identifies 535 new loci associated with blood pressure traits. Nature genetics. 2018;50(10):1412.

13. Warren HR, Evangelou E, Cabrera CP, Gao H, Ren M, Mifsud B, et al. Genome-wide association analysis identifies novel blood pressure loci and offers biological insights into cardiovascular risk. Nature genetics. 2017;49(3):403.

14. Furberg H, Kim Y, Dackor J, Boerwinkle E, Franceschini N, Ardissino D, et al. Genome-wide meta-analyses identify multiple loci associated with smoking behavior. Nature genetics. 2010;42(5):441.

15. Wootton RE, Richmond RC, Stuijfzand BG, Lawn RB, Sallis HM, Taylor GMJ, et al. Causal effects of lifetime smoking on risk for depression and schizophrenia: Evidence from a Mendelian randomisation study. bioRxiv. 2018:381301.

16. Willer CJ, Schmidt EM, Sengupta S, Peloso GM, Gustafsson S, Kanoni S, et al. Discovery and refinement of loci associated with lipid levels. Nat Genet. 2013;45(11):1274-83.

17. Klarin D, Damrauer SM, Cho K, Sun YV, Teslovich TM, Honerlaw J, et al. Genetics of blood lipids among~ 300,000 multi-ethnic participants of the Million Veteran Program. Nature genetics. 2018;50(11):1514.

18. Wood AR, Esko T, Yang J, Vedantam S, Pers TH, Gustafsson S, et al. Defining the role of common variation in the genomic and biological architecture of adult human height. Nature genetics. 2014;46(11):1173.

19. Locke AE, Kahali B, Berndt SI, Justice AE, Pers TH, Day FR, et al. Genetic studies of body mass index yield new insights for obesity biology. Nature. 2015;518(7538):197.

20. Speliotes EK, Willer CJ, Berndt SI, Monda KL, Thorleifsson G, Jackson AU, et al. Association analyses of 249,796 individuals reveal 18 new loci associated with body mass index. Nature genetics. 2010;42(11):937.

21. Burgess S, Thompson SG. Multivariable Mendelian randomization: the use of pleiotropic genetic variants to estimate causal effects. American journal of epidemiology. 2015;181(4):251-60.

22. Burgess S, Butterworth A, Thompson SG. Mendelian randomization analysis with multiple genetic variants using summarized data. Genetic epidemiology. 2013;37(7):658-65.

23. Zheng X, Levine D, Shen J, Gogarten SM, Laurie C, Weir BS. A high-performance computing toolset for relatedness and principal component analysis of SNP data. Bioinformatics. 2012;28(24):3326-8.

24. Bowden J, Davey Smith G, Haycock PC, Burgess S. Consistent estimation in Mendelian randomization with some invalid instruments using a weighted median estimator. Genetic epidemiology. 2016;40(4):304-14.

25. Bowden J, Davey Smith G, Burgess S. Mendelian randomization with invalid instruments: effect estimation and bias detection through Egger regression. International journal of epidemiology. 2015;44(2):512-25.

26. Yavorska OO, Burgess S. MendelianRandomization: an R package for performing Mendelian randomization analyses using summarized data. International journal of epidemiology. 2017;46(6):1734-9.

27. de Leeuw CA, Mooij JM, Heskes T, Posthuma D. MAGMA: generalized gene-set analysis of GWAS data. PLoS Comput Biol. 2015;11(4):e1004219.

28. Watanabe K, Taskesen E, Van Bochoven A, Posthuma D. Functional mapping and annotation of genetic associations with FUMA. Nature communications. 2017;8(1):1-11.

29. Bulik-Sullivan BK, Loh P-R, Finucane HK, Ripke S, Yang J, Patterson N, et al. LD Score regression distinguishes confounding from polygenicity in genome-wide association studies. Nature genetics. 2015;47(3):291.

30. Pasaniuc B, Price AL. Dissecting the genetics of complex traits using summary association statistics. Nature Reviews Genetics. 2017;18(2):117-27.

31. Klarin D, Verma SS, Judy R, Dikilitas O, Wolford BN, Paranjpe I, et al. Genetic architecture of abdominal aortic aneurysm in the million veteran program. Circulation. 2020;142(17):1633-46.

32. van der Harst P, Verweij N. Identification of 64 novel genetic loci provides an expanded view on the genetic architecture of coronary artery disease. Circulation research. 2018;122(3):433-43.

33. Malik R, Chauhan G, Traylor M, Sargurupremraj M, Okada Y, Mishra A, et al. Multiancestry genome-wide association study of 520,000 subjects identifies 32 loci associated with stroke and stroke subtypes. Nature genetics. 2018;50(4):524-37.

34. Bulik-Sullivan B, Finucane HK, Anttila V, Gusev A, Day FR, Loh P-R, et al. An atlas of genetic correlations across human diseases and traits. Nature genetics. 2015;47(11):1236.
